# Supplementary figures and images for: Dopamine and its receptor DcDop2 are involved in the coevolution between ‘Candidatus Liberibacter asiaticus’ and Diaphorina citri
Source: eLife. 2026 Jun 22;15:RP109081. doi: 10.7554/eLife.109081 (PMC13286569; doi:10.7554/eLife.109081)

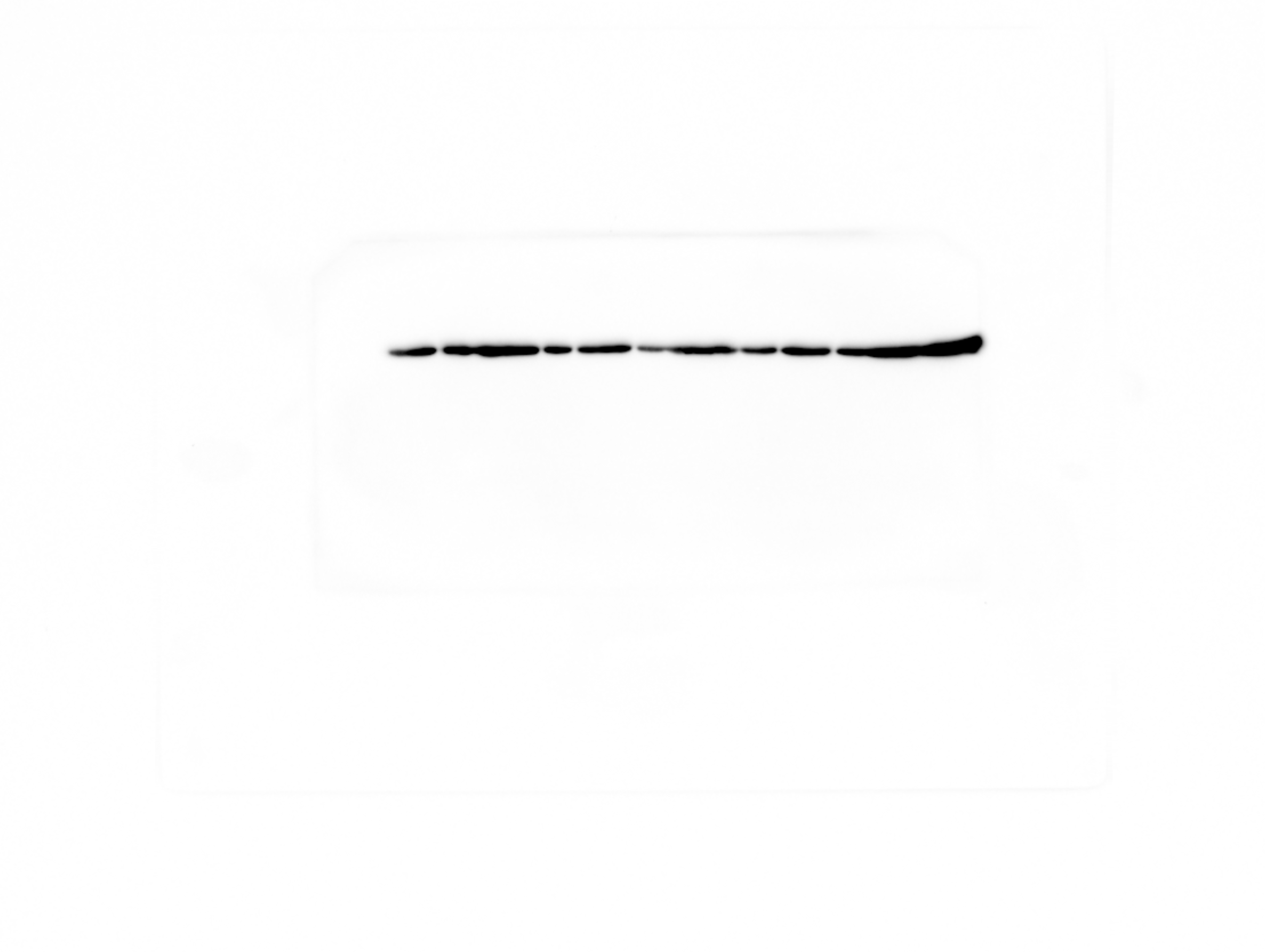

Supplement: Figure 3—source data 1. [file elife-109081-fig3-data1.zip › Figure 3B DcDop2 original.tif]

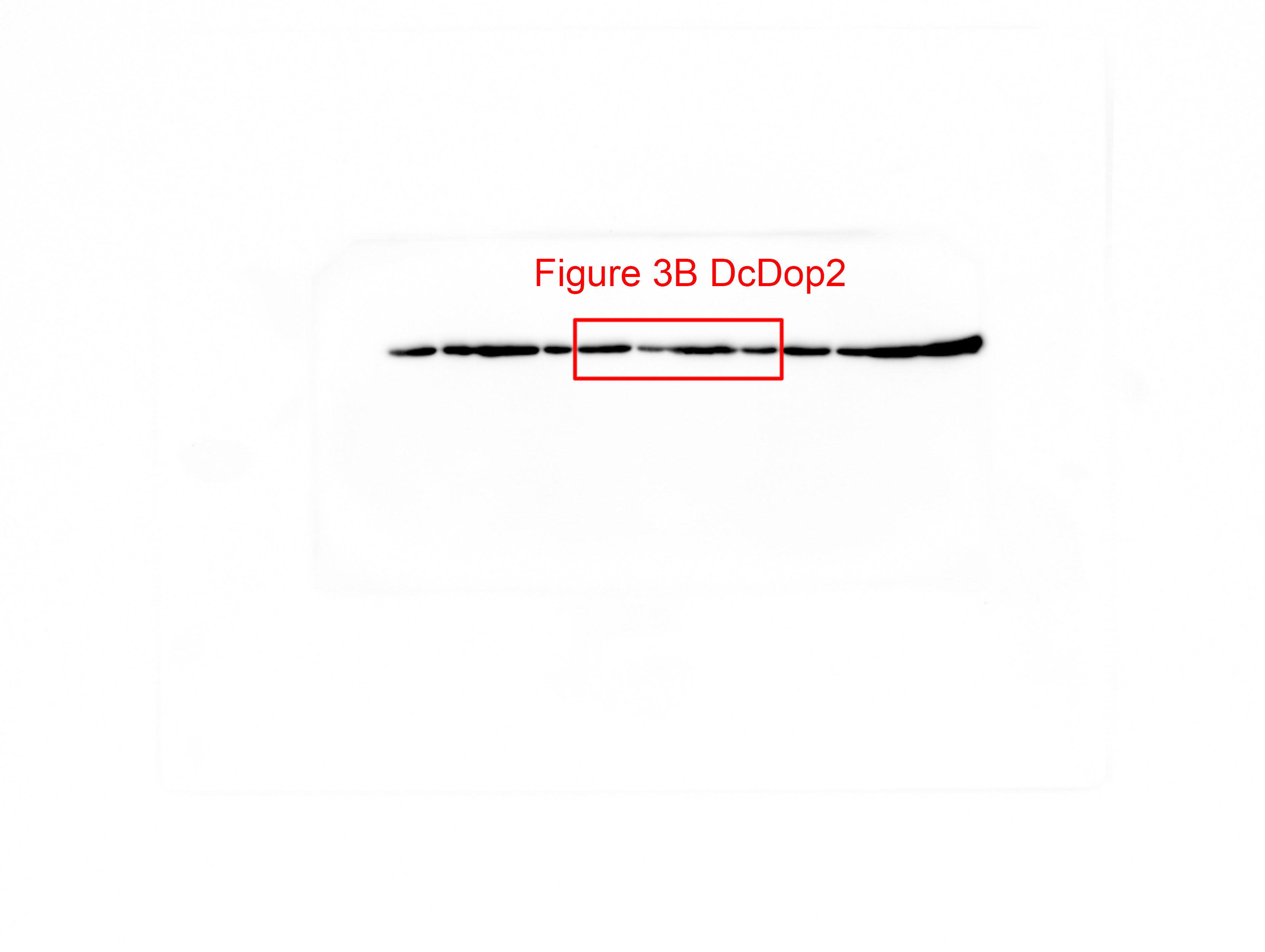

Supplement: Figure 3—source data 1. [file elife-109081-fig3-data1.zip › Figure 3B DcDop2 labelled.jpg]

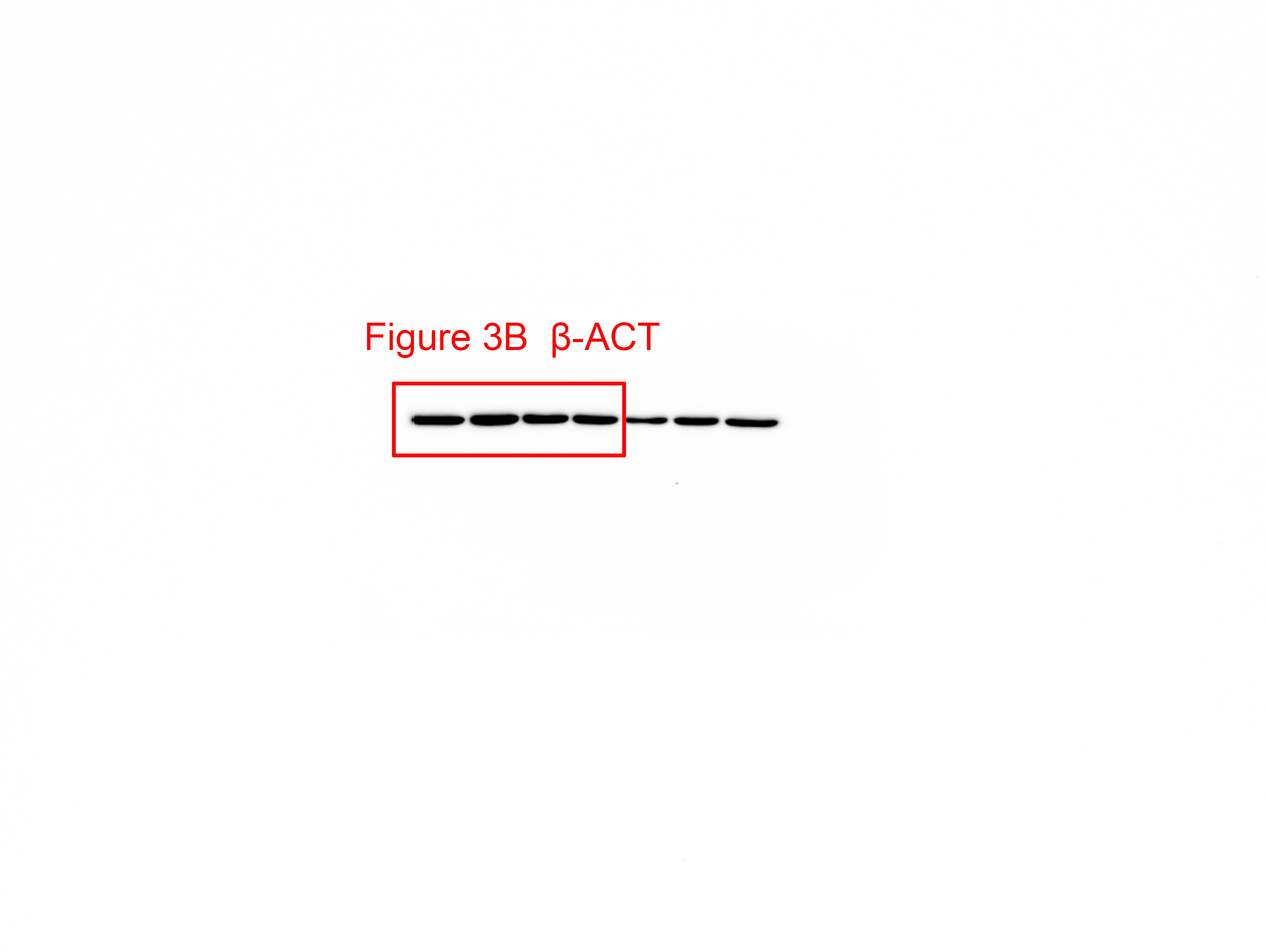

Supplement: Figure 3—source data 2. [file elife-109081-fig3-data2.zip › Figure 3B ╬▓-ACT labelled.tif]

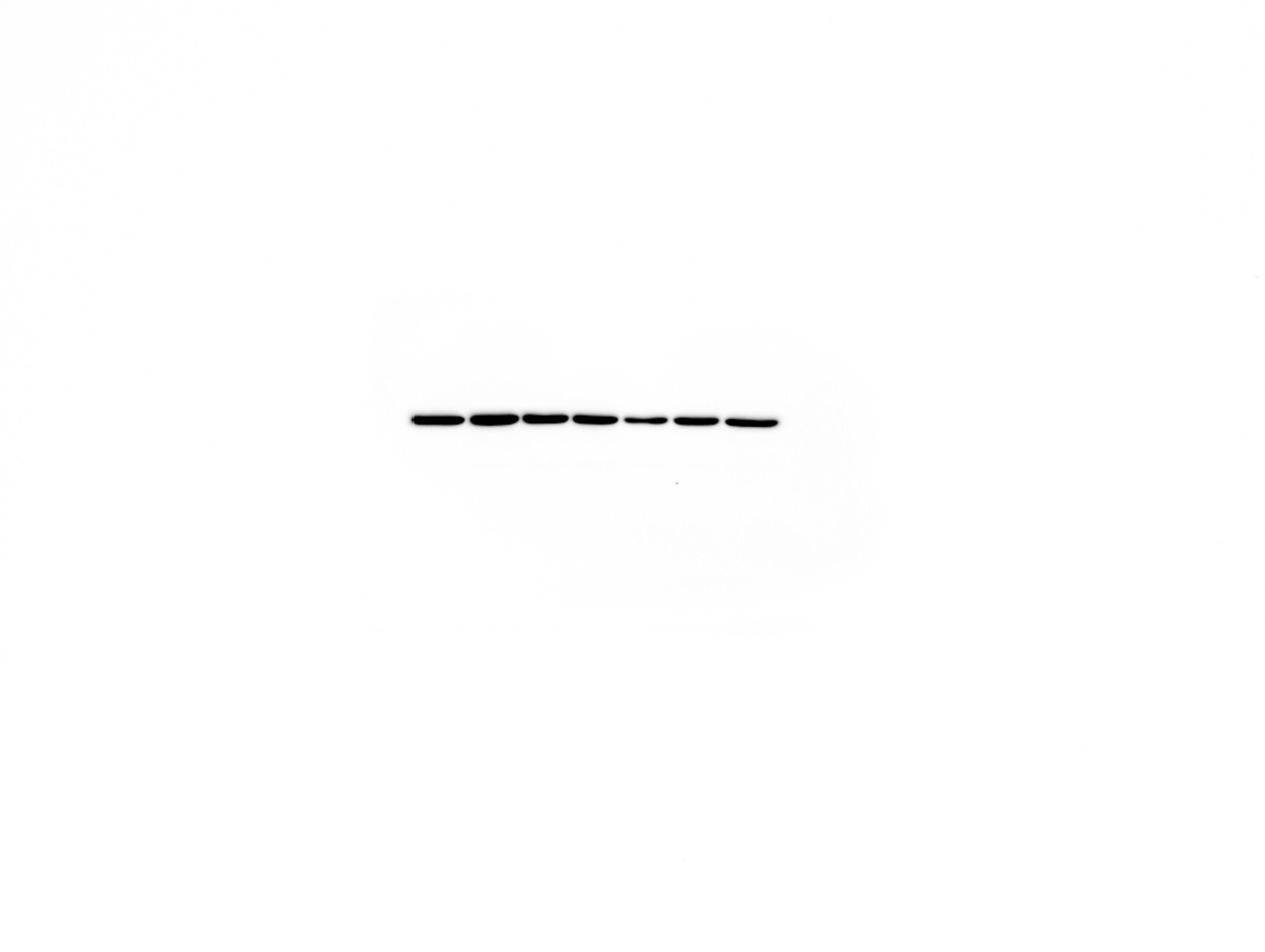

Supplement: Figure 3—source data 2. [file elife-109081-fig3-data2.zip › Figure 3B-╬▓-ACT original.tif]

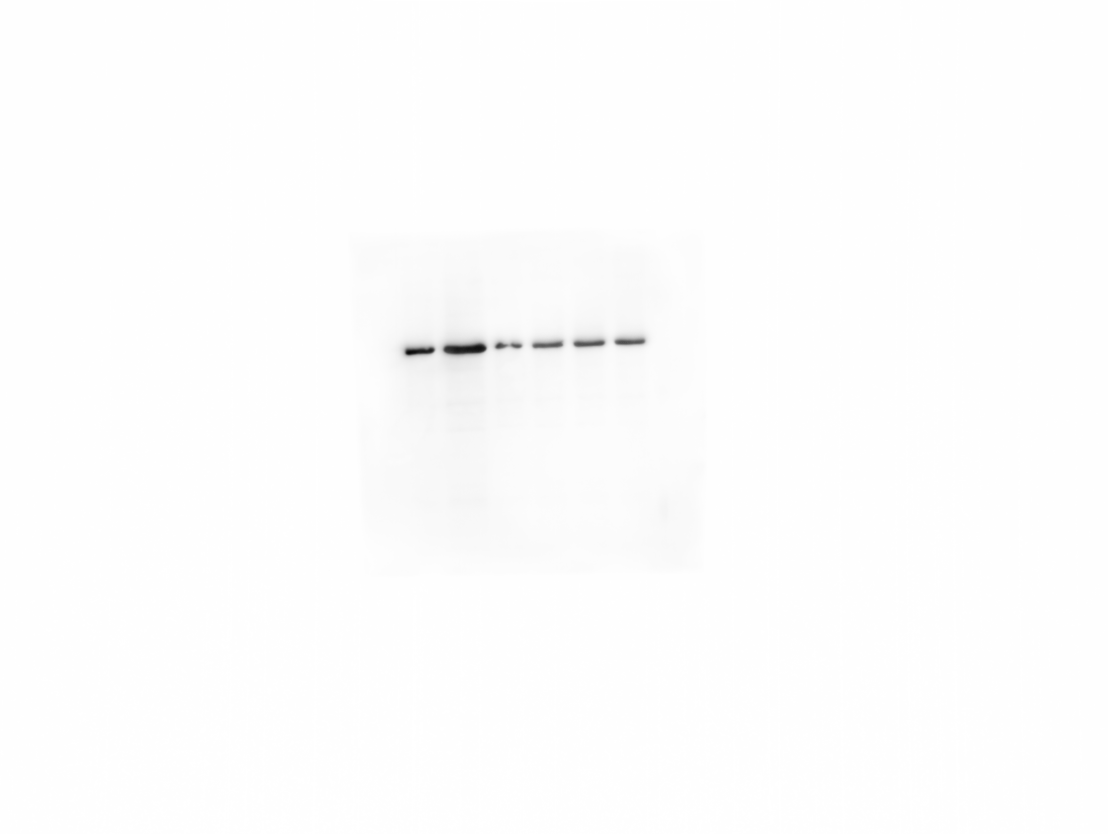

Supplement: Figure 4—source data 1. [file elife-109081-fig4-data1.zip › Figure 4G DcDop2 original.tif]

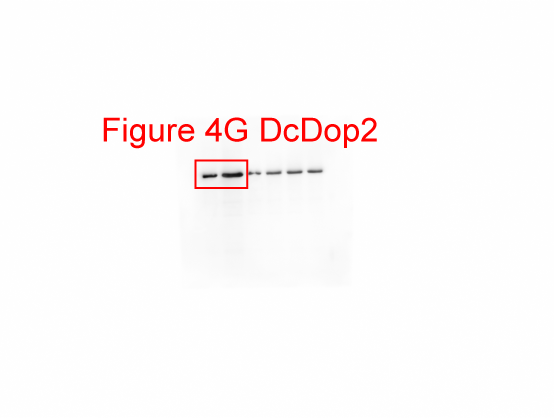

Supplement: Figure 4—source data 1. [file elife-109081-fig4-data1.zip › Figure 4G DcDop2 labelled.tif]

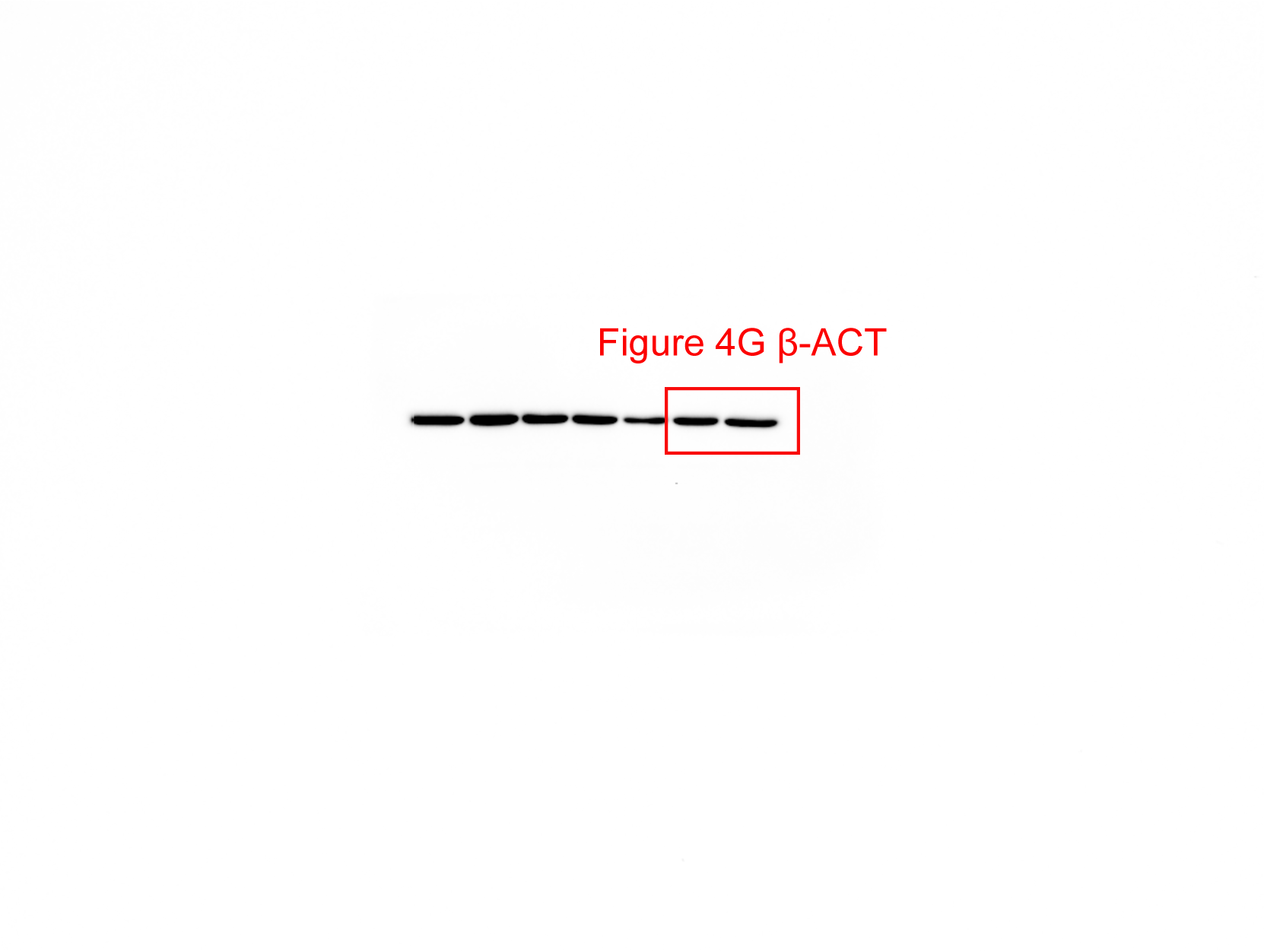

Supplement: Figure 4—source data 2. [file elife-109081-fig4-data2.zip › Figure 4G ╬▓-ACT labelled.tif]
