# Supplementary material for: Dopamine and its receptor DcDop2 are involved in the coevolution between ‘Candidatus Liberibacter asiaticus’ and Diaphorina citri
Source: eLife. 2026 Jun 22;15:RP109081. doi: 10.7554/eLife.109081 (PMC13286569; doi:10.7554/eLife.109081)
Supplement: Supplementary file 1. [file elife-109081-supp1.docx]

**Supplementary File 1. The primers used in this study.**

| **Gene**  **name** | **Accession number** | **primer name** | **Sequences of primers (5’-3’)** | **Product size (bp)** | **Purpose** |
| --- | --- | --- | --- | --- | --- |
| *DcDdc* | XM_008486080.3 | *DcDdc*-Full-F | CGAACTCGTTTTGTTGTT | 1499 | Validation of full-length sequence |
|  |  | *DcDdc*-Full-R | AGCTTTTTCCTTTTCCTCCT |  |  |
|  |  | *DcDdc*-qF | ATGACAGGTTTGAGGTCATC | 103 | qRT-PCR |
|  |  | *DcDdc*-qR | CCGTTGATACGTTTGAGAAG |  |  |
|  |  | *DcDdc*-RNAi-F | taatacgactcactatagggATAGACGCGTTTTACCGACC | 424 | dsRNA synthesis |
|  |  | *DcDdc*-RNAi-R | taatacgactcactatagggACTCTCTGCATGGTTTTGGC |  |  |
| *DcDop2* | XM_026822145.1 | *DcDop2*-Full-F1 | ACGGAATGTGAACTCAGGAT | 962 | Validation of full-length sequence |
|  |  | *DcDop2*-Full-R1 | TGGTGTATGGTGCAATGT |  |  |
|  |  | *DcDop2*-Full-F2 | CCCTTTCACTGATAGCAA | 1333 |  |
|  |  | *DcDop2*-Full-R2 | CTCGCTTTAGATTCCACATG |  |  |
|  |  | *DcDop2*-3UTR-F | GATGAATCCTGTCATCTACGC | 435 | 3’UTR amplification |
|  |  | 3’RACE-Outer Primer | TACCGTCGTTCCACTAGTGATTT |  |  |
|  |  | *DcDop2*-qF | TGCTTCTCCAGGAAATATCG | 124 | qRT-PCR |
|  |  | *DcDop2*-qR | ACTGGTTGACTGTGGAATAC |  |  |
|  |  | *DcDop2*-RNAi-F | taatacgactcactatagggTACGAAGCAAGTGATGTCGG | 493 | dsRNA synthesis |
|  |  | *DcDop2*-RNAi-R | taatacgactcactatagggGAAGCAGGAGCACAAGATCC |  |  |
|  |  | *DcDop2*-3’UTR-Full-F | ctagttgtttaaacgagctTTCGACCGGAGTTTCCA | 191 | Full sequence of 3’UTR |
|  |  | *DcDop2*-3’UTR-Full-R | tgcatgcctgcaggtcgactctagaTCAAGTCCACGATTCTTAC |  |  |
|  |  | *DcDop2*-3’UTR-mutant-F | ctagttgtttaaacgagctcGTCTTTTAACAGCAATATG | 136 | Mutant sequence of 3’UTR |
|  |  | *DcDop2*-3’UTR-mutant-R | tgcatgcctgcaggtcgactctagaTCAAGTCCACGATTCTTAC |  |  |
|  |  | *DcDop2*-probe | FAM-TGTGTCTACGCCGATATTTC | - | Labeled with FAM for FISH |
| *GFP* | ACY56286 | GFP-RNAi-F | taatacgactcactatagggACTCCAGCAGGACCATGTGATC | 596 | dsRNA synthesis |
|  |  | GFP-RNAi-R | taatacgactcactatagggACCTGAAGTTCATCTGCACCAC |  |  |
| *Dcβ-ACT* | DQ675553.1 | β-ACT-qF | TGTTCCAACCTTCCTTCCTG | 109 | qRT-PCR |
|  |  | β-ACT-qR | GTGTTGGCGTACAGGTCCTT |  |  |
| *DcAKH* | MG550150.1 | *DcAKH*-qF | TGGACTCTCTCAAGTACATTT | 54 | qRT-PCR |
|  |  | *DcAKH*-qR | ACAATTTTTGGGCTTCACTC |  |  |
| *DcAKHR* | OR259432 | *DcAKHR*-qF | TCAGTTGGAAGAAGGACAAG | 116 | qRT-PCR |
|  |  | *DcAKHR*-qR | GGTACTCAGGCTACACTCTA |  |  |
| *DcMet* | OP251123 | *DcMet*-qF | AAGCTCAAGGGCCAAGTCAT | 257 | qRT-PCR |
|  |  | *DcMet*-qR | TAATCAATACCAGGGGCGGC |  |  |
| *DcKr-h1* | XM_026820026.1 | *DcKr-h1-qF* | CTCCAGTGCTGAGTCCACAA | 103 | qRT-PCR |
|  |  | *DcKr-h1-qR* | ATCTCCCGGAGGTTTCTGTT |  |  |
| *DcVg-1-like* | XM_008488883.3 | *DcVg-1-like-qF* | CACCTACTCCTTGTCCTCTA | 166 | qRT-PCR |
|  |  | *DcVg-1-like-qR* | GAAAAATCCCCAGAGTCCTT |  |  |
| *DcVg-A1-like* | XM_026832896.1 | *DcVg-A1-like-qF* | CTCCTCAGAAAGTGGAAGTT | 132 | qRT-PCR |
|  |  | *DcVg-A1-like-qR* | TTGTTTCCGATGAAGTAGGG |  |  |
| *DcVgR* | OP251122 | *DcVgR-qF* | AGCAGCTGGATATACATGTG | 186 | qRT-PCR |
|  |  | *DcVgR-qR* | CTCCACAGTACTGATTACCG |  |  |
| *C*Las 16s rRNA | L22532.1 | *C*Las 16s-probe | Cy3-CATTATCTTCTCCGGCG | - | Labeled with Cy3 for FISH |
|  |  | *C*Las 16s-qF | TCGAGCGCGTATGCAATACG |  | qRT-PCR |
|  |  | *C*Las 16s-qR | GCGTTATCCCGTAGAAAAAGGTAG |  |  |
|  |  | *C*Las 16s-Actin-qF | CCCTGGACTTTGAACAGGAA |  |  |
|  |  | *C*Las 16s-Actin-qR | CTCGTGGATACCGCAAGATT |  |  |
| miR-31a | - | miR-31a-qF | TGGCAAGATGTCGGCATAGCTGA |  | qRT-PCR |
|  |  | miR-31a-probe | Cy3-TCAGCTATGCCGACATCTTGCCA | - | Labeled with Cy3 for FISH |
| U6 | - | U6-qF | AGGATGACACGCAAAATCGT | - | qRT-PCR |
| *DcDop1* | XM_017446452.2 | *DcDop1*-qF | ATACGTGCTGTCTGTGTTAC | 177 | qRT-PCR |
|  |  | *DcDop1*-qR | GCACGTAGTCCGAATAGTTA |  |  |
| *DcDop3* | XM_008472271.3 | *DcDop3*-qF | ATTCTCATTATCGCTGGGTC | 110 | qRT-PCR |
|  |  | *DcDop3*-qR | GGTGGTGAAAATGAACCAAG |  |  |
| *DcTh* | XM_017442547.2 | *DcTh*-qF | GAAAAATTCAGACGTTGGGT | 126 | qRT-PCR |
|  |  | *DcTh*-qR | GTTGAGTTGGGACATAAGGT |  |  |
|  |  | *DcTh*-RNAi-F | taatacgactcactatagggCACCTAGCGACAACCAATCA | 498 | dsRNA synthesis |
|  |  | *DcTh*-RNAi-R | taatacgactcactatagggATGTCCTTTCGTCGCTGTCT |  |  |
| *DcHenna2* | XM_008489662.2 | *Dchenna2*-qF | CTTGTACTTTGTGGCTGAGA | 176 | qRT-PCR |
|  |  | *Dchenna2*-qR | TCTGCATTTCCCCGTTAATA |  |  |
| DcHenna1 | XM_017449691.2 | *Dchenna1*-qF | TCAGGAAGGTCAACTCAAAG | 167 | qRT-PCR |
|  |  | *Dchenna1*-qR | TCTCAGCCACAAAGTACAAG |  |  |
|  |  | *Dchenna1-*RNAi-F | taatacgactcactatagggTGGTGGTGACTTGTCCAGTG | 468 | dsRNA synthesis |
|  |  | *Dchenna1*-RNAi-R | taatacgactcactatagggCGCAAGGAGAACCCTGTACT |  |  |
| *DcVat2* | XM_008471681.2 | *DcVat2*-qF | CTCCGCAGAGGTTAAATGA | 184 | qRT-PCR |
|  |  | *DcVat2*-qR | TGCTGCAATTTCTCCACTAA |  |  |
| *DcVat1* | XM_026831608.1 | *DcVat1*-qF | AGTTTAGAGCCAAAACCCAA | 147 | qRT-PCR |
|  |  | *DcVat1*-qR | GGCTTCTTTTGTATCTGGTG |  |  |
|  |  | *DcVat1-*RNAi-F | taatacgactcactatagggCAGGAGGGTGCAGCTCTTAC | 498 | dsRNA synthesis |
|  |  | *DcVat1*-RNAi-R | taatacgactcactatagggGAAGTGGCGCAAATTGAATC |  |  |

Note: The lowercase with underline indicated the T7 promoter sequences in the primers of dsRNA synthesis. The sequences with black boxes displayed the homologous arm sequence used for vector seamless clone.
