# Supplementary material for: Dopamine and its receptor DcDop2 are involved in the coevolution between ‘Candidatus Liberibacter asiaticus’ and Diaphorina citri
Source: eLife. 2026 Jun 22;15:RP109081. doi: 10.7554/eLife.109081 (PMC13286569; doi:10.7554/eLife.109081)
Supplement: Peer review history

# Editor report: eLife Assessment

**DOI:** 10.7554/eLife.109081.3.sa0

**Authors:** Kirsten Pelz-Stelinski

**Affiliations:** University of Florida United States

**Keywords:** Convincing, Important

Insects can act as vectors of plant diseases; hence, the study of insect–pathogen interactions is relevant for agriculture. This **important** study identifies in *Diaphorina citri* a dopamine receptor responsive to ‘*Candidatus* Liberibacter asiaticus’ infection, demonstrates direct regulation of this receptor by a microRNA, and integrates dopamine signaling into an established insect reproductive hormone framework. Multiple complementary experimental approaches **convincingly** support the findings, although key conclusions rely on correlative data and the mechanistic evidence for the proposed linear signaling cascade is limited. This work will be of interest for insect physiology and vector–pathogen biology, and more broadly for citrus agriculture.

# Referee report: Reviewer #1 (Public review)

**DOI:** 10.7554/eLife.109081.3.sa1

I read this paper with great interest based on my experience in insect sciences. Previous concerns:

(1) The paper has an original biological question that is overly broad and mechanistically ambitious. The central biological question, namely how CLas infection enhances fecundity of Diaphorina citri via dopamine signaling, is clearly stated and well motivated by previous literature. However, my advice to the authors is that, while the general question is clear, the manuscript attempts to answer multiple mechanistic layers simultaneously. As a result, I feel that the biological narrative becomes diffuse, especially in later sections where DA, miRNA regulation, AKH signaling, and JH signaling are all proposed as parts of a single linear cascade. In summary, my key concern is that the paper often moves from correlation to causal hierarchy without fully disentangling whether these pathways act sequentially, in parallel, or redundantly. A more explicitly framed primary hypothesis (e.g., "DA-DcDop2 is necessary and sufficient for CLas-induced fecundity") may improve conceptual clarity.

(2) On the novelty of the data, I feel they are moderately novel, with substantial confirmatory components. If I am correct, the novel contributions include the identification of DcDop2 as the DA receptor responsive to CLas infection in D. citri, the discovery that miR-31a directly targets DcDop2, which is supported by luciferase assays and RIP, and thirdly, the integration of dopamine signaling into the already-described CLas-AKH-JH-fecundity framework. My advice to the authors is to focus more on the manuscript's novelty, which lies more in pathway integration than in discovering fundamentally new biological phenomena. This is appropriate for a mechanistic paper, but should be framed as an extension of existing models rather than a paradigm shift.

(3) On the conclusions, I recommend that the authors modify their statements a little. I feel that there are some overstated or insufficiently supported claims. For instance, the assertion that CLas "hijacks" the DA-DcDop2-miR-31a-AKH-JH cascade implies direct pathogen manipulation, but no CLas-derived effector or mechanism is identified. Also, that the model suggests a linear signaling hierarchy, but the data largely show correlation and partial dependency rather than strict epistasis. In third, the term "mutualistic interaction" may be too strong, as host fitness costs outside fecundity (e.g., longevity, immunity) are not evaluated. In conclusion, I confirm that the data support a functional association, but mechanistic causality and evolutionary interpretation are somewhat overstated.

Comments on revised version:

The authors provided a satisfactory revision.

# Referee report: Reviewer #2 (Public review)

**DOI:** 10.7554/eLife.109081.3.sa2

Summary:

Nian and colleagues comprehensively apply metabolomics, molecular, and genetic approaches to demonstrate that CLas hijacks the DA/DcDop2-miR-31a-AKH-JH signaling cascade to enhance lipid metabolism and fecundity in D. citri, while concurrently promoting its own replication.

Strengths:

These findings provide solid evidence of a mutualistic interaction between CLas proliferation and ovarian development in the insect host. This insight significantly advances our understanding of the molecular interplay between plant pathogens and vector insects and offers novel targets and strategies for HLB field management.

Weaknesses:

While the article investigates the involvement of dopamine signaling and specific microRNAs in enhancing fecundity and pathogen proliferation, it still needs to provide a detailed mechanistic understanding of these interactions. The precise molecular pathways and feedback mechanisms by which CLas manipulates dopamine signaling in Diaphorina citri remain unclear.

# Author comment: Author response

**DOI:** 10.7554/eLife.109081.3.sa3

**Authors:** Xiaoge Nian, Jiayun Li, Jilei Huang, Weiwei Yuan, Paul Holford, George Andrew Charles Beattie, Jielan He, Yijing Cen, Yurong He, Songdou Zhang

**Affiliations:** South China Agricultural University Guangzhou China; South China Agricultural University Guangzhou China; South China Agricultural University Guangzhou China; South China Agricultural University Guangzhou China; Western Sydney University Penrith Australia; Western Sydney University Penrith Australia; South China Agricultural University Guangzhou China; South China Agricultural University Guangzhou China; South China Agricultural University Guangzhou China; Guangdong Academy of Agricultural Sciences Guangzhou China

The following is the authors’ response to the original reviews.

**Public Reviews:**

**Reviewer #1 (Public review):**

I read this paper with great interest based on my experience in insect sciences. I have some minor comments (and recommendations) that I believe the authors should address.

(1) The paper has an original biological question that is overly broad and mechanistically ambitious. The central biological question, namely how CLas infection enhances fecundity of *Diaphorina citri* via dopamine signaling, is clearly stated and well motivated by previous literature. However, my advice to the authors is that, while the general question is clear, the manuscript attempts to answer multiple mechanistic layers simultaneously. As a result, I feel that the biological narrative becomes diffuse, especially in later sections where DA, miRNA regulation, AKH signaling, and JH signaling are all proposed as parts of a single linear cascade. In summary, my key concern is that the paper often moves from correlation to causal hierarchy without fully disentangling whether these pathways act sequentially, in parallel, or redundantly. A more explicitly framed primary hypothesis (e.g., "DA-DcDop2 is necessary and sufficient for CLas-induced fecundity") may improve conceptual clarity.

We sincerely thank the reviewer for these constructive comments and agreed that the initial version of our manuscript attempted to integrate multiple signaling layers, which may have blurred the logical distinction between sequential, parallel, or redundant pathways. To address this concern, we have restructured the narrative to center on a clearly defined hypothesis by changing “DA/*DcDop2*-miR-31a-AKH-JH signaling cascade” to “DA-*DcDop2* signaling axis” in Abstract (Line 33) of the revised manuscript.

(2) On the novelty of the data, I feel they are moderately novel, with substantial confirmatory components. If I am correct, the novel contributions include the identification of *DcDop2* as the DA receptor responsive to CLas infection in *D. citri*, the discovery that miR-31a directly targets *DcDop2*, which is supported by luciferase assays and RIP, and thirdly, the integration of dopamine signaling into the already-described CLas-AKH-JH-fecundity framework. My advice to the authors is to focus more on the manuscript's novelty, which lies more in pathway integration than in discovering fundamentally new biological phenomena. This is appropriate for a mechanistic paper, but should be framed as an extension of existing models rather than a paradigm shift.

We sincerely thank the reviewer for this thoughtful and highly constructive assessment. We greatly appreciate the clear articulation of what constitutes the novel contributions of our work, and we fully agree with the characterization that the primary novelty lies in pathway integration rather than the discovery of entirely unprecedented biological phenomena. We also accept the valuable advice that our manuscript should be framed as an extension of existing models rather than a paradigm shift. In response to this insightful comment, we have carefully revised the Results part in Line 275-278 of the revised manuscript.

(3) On the conclusions, I recommend that the authors modify their statements a little. I feel that there are some overstated or insufficiently supported claims. For instance, the assertion that CLas "hijacks" the DA-*DcDop2*-miR-31a-AKH-JH cascade implies direct pathogen manipulation, but no CLas-derived effector or mechanism is identified. Also, that the model suggests a linear signaling hierarchy, but the data largely show correlation and partial dependency rather than strict epistasis. In third, the term "mutualistic interaction" may be too strong, as host fitness costs outside fecundity (e.g., longevity, immunity) are not evaluated. In conclusion, I confirm that the data support a functional association, but mechanistic causality and evolutionary interpretation are somewhat overstated.

We sincerely thank the reviewer for these insightful comments and agreed that there are some overstated or insufficiently supported claims. In response to this insightful comment, we have changed "hijacks" to "regulates" (Line 32 and 124), and "mutualistic interaction" to “coevolution” (Line 2, 34, 127, 257, 763, 806, and 842) in our revised manuscript.

**Reviewer #2 (Public review):**

Summary:

Nian and colleagues comprehensively apply metabolomics, molecular, and genetic approaches to demonstrate that CLas hijacks the DA/DcDop2-miR-31a-AKH-JH signaling cascade to enhance lipid metabolism and fecundity in D. citri, while concurrently promoting its own replication.

Strengths:

These findings provide solid evidence of a mutualistic interaction between CLas proliferation and ovarian development in the insect host. This insight significantly advances our understanding of the molecular interplay between plant pathogens and vector insects, and offers novel targets and strategies for HLB field management.

Weaknesses:

While the article investigates the involvement of dopamine signaling and specific microRNAs in enhancing fecundity and pathogen proliferation, it still needs to provide a detailed mechanistic understanding of these interactions. The precise molecular pathways and feedback mechanisms by which CLas manipulates dopamine signaling in Diaphorina citri remain unclear.

These comments are extremely helpful for revising and improving our manuscript.

**Recommendations for the authors:**

**Reviewer #2 (Recommendations for the authors):**

(1) In Figures 1C and 1D, please maintain consistent gene nomenclature: change "henna" to "Henna", "TH" to "Th", and "DDC" to "Ddc".

Thanks for your great suggestion. We have changed "henna" to "Henna", "TH" to "Th", and "DDC" to "Ddc" in Figure 1C and 1D of our revised manuscript.

(2) In Figure 7, correct "Emergy metabolism" to "Energy metabolism".

Thanks for your valuable suggestion. We have corrected "Emergy metabolism" to "Energy metabolism" in Figure 7 of our revised manuscript.

(3) Please specify the number of biological replicates in the figure captions.

Thanks for your perfect suggestion. We have specified the number of biological replicates in the figure captions of Figure 1 (Line 737-738), Figure 2 (Line 757-759), Figure 3 (Line 780-782), Figure 4 (Line 799-800), Figure 5 (Line 816-819), and Figure 6 (Line 833-836).

(4) For Figure 2I, 3J, and 5H, clarify that CLas 16s rRNA was detected by FISH. The age of the dissected females should also be described in the captions.

Thanks for your insightful suggestion. We have added the female age (at 7 DAE) in the captions for Figure 2I (Line 752), 3J (Line 773), and 5H (Line 813) of our revised manuscript.

(5) A blot is shown in Figure 3B but not discussed in the text. Since the manuscript describes mRNA levels, please specify whether these blots are from Northern or Western blotting and provide relevant methodological details.

Thanks for your great suggestion. The blot in Figure 3B is Western blot result. We have added the related descriptions in Result (Line 202), Materials and Methods (Line 521-536), and figure legend (Line 766) of our revised manuscript.

(6) In Figure 3G-3K, an "inhibitor" was used, but its name and functional role are not described. Please give more details.

Thanks for your valuable suggestion. We have added the detail information for “Dop2 inhibitor” in the Figure 3G-3K legend (Line 772-776) of our revised manuscript.

(7) In Lines 23-24 of the Abstract, consider revising "their neuroendocrine regulation remains unclear" to "their neuroendocrine regulation mechanisms remain unclear" for grammatical accuracy.

Thanks for your perfect suggestion. We have revised "their neuroendocrine regulation remains unclear" to "their neuroendocrine regulation mechanisms remain unclear" for grammatical accuracy in Line 24 of our revised manuscript.

(8) The last sentence of the Abstract is overly long. It is recommended to split it as follows: "These findings reveal a mutualistic interaction between CLas proliferation and ovarian development in the insect host. This discovery enhances our understanding of the molecular interplay between plant pathogens and vector insects and offers novel targets and strategies for HLB field management."

Thanks for your excellent suggestion. We have splited the last sentence of the Abstract as follows: "These findings reveal a coevolution between CLas proliferation and ovarian development in the insect host. This discovery enhances our understanding of the molecular interplay between plant pathogens and vector insects and offers novel targets and strategies for HLB field management." in Line 34-37 of our revised manuscript.

(9) In Line 139, remove the comma between "female" and "adult".

Thanks for your great suggestion. We have removed the comma between "female" and "adult" in Line 139 of our revised manuscript.

(10) In Line 149, replace "d" with day.

Thanks for your perfect suggestion. We have replaced "d" with "day" in Line 149 of our revised manuscript.

(11) The JH determination method references a previous study but lacks a detailed description of the extraction procedure. Please include this information in the methodology section.

Thanks for your valuable suggestion. We have added the detailed description of the JH extraction procedure in Line 511-514 of our revised manuscript.

(12) In Figure S2, since the panel shows interference efficiencies for four genes, "treated with dsDcAKHR" should be revised to "treated with dsRNA" for accuracy.

Thanks for your insightful suggestion. We have revised "treated with dsDcAKHR" to "treated with dsRNA" for accuracy in the Figure S2 legend.

(13) In line 354-355, change "DcVg1-like, DcVgA1-like and DcVgR" to "DcVg1-like, DcVgA1-like, and DcVgR".

Thanks for your great suggestion. We have changed "*DcVg1-like*, *DcVgA1-like* and *DcVgR*" to "*DcVg1-like*, *DcVgA1-like*, and *DcVgR*" in Line 350 of our revised manuscript.

(14) The study primarily investigates the role of agomir-31a. Would antagomir-31a promote ovarian development in CLas- females? In addition, did the authors perform a rescue experiment using antagomir-31a in CLas+ females after dsDcDop2 treatment?

Thanks for your valuable suggestion. The proposed experiments will be instrumental in further elucidating the functional role of miR-31a and represent a key direction for our future research. We will carefully consider and incorporate these approaches in our subsequent study.

(15) The method used to determine CLas-negative and CLas-positive individuals should be described in more detail in the Materials and Methods section.

Thanks for your great suggestion. We have added more details about *C*Las detection in the Materials and Methods section (Line 378) of our revised manuscript.
